# A New Approach to Virtual Occlusion in Orthognathic Surgery Planning Using Mixed Reality—A Technical Note and Review of the Literature

**DOI:** 10.3390/jpm13121709

**Published:** 2023-12-14

**Authors:** Max Wilkat, Shufang Liu, Michael Schwerter, Felix Schrader, Leonardo Saigo, Nadia Karnatz, Norbert R. Kübler, Majeed Rana

**Affiliations:** 1 Department of Oral and Plastic Maxillofacial Surgery, Heinrich Heine University Hospital Düsseldorf, Moorenstraße 5, 40225 Düsseldorf, Germany; 2 Brainlab AG, Olof-Palme-Str. 9, 81829 München, Germany; 3 Department of Oral and Maxillofacial Surgery, National Dental Centre Singapore, 5 Second Hospital Ave., Singapore 168938, Singapore

**Keywords:** virtual occlusion, orthognathic surgery, computer-assisted planning, mixed reality, intra-oral scanning

## Abstract

Orthognathic surgery plays a vital role in correcting various skeletal discrepancies of the maxillofacial region. Achieving optimal occlusion is a fundamental aspect of orthognathic surgery planning, as it directly influences postoperative outcomes and patient satisfaction. Traditional methods for setting final occlusion involve the use of dental casts which are time-consuming, prone to errors and cannot be easily shared among collaborating specialties. In recent years, advancements in digital technology have introduced innovative approaches, such as virtual occlusion, which may offer enhanced accuracy and efficiency in orthognathic surgery planning. Furthermore, the emergence of mixed reality devices and their 3D visualization capabilities have brought about novel benefits in the medical field, particularly in computer-assisted planning. This paper presents for the first time a prototype tool for setting virtual occlusion during orthognathic surgery planning using mixed reality technology. A complete walkthrough of the workflow is presented including an explanation of the implicit advantages of this novel tool. The new approach to defining virtual occlusion is set into context with other published methods of virtual occlusion setting, discussing advantages and limitations as well as concepts of surgical occlusion for orthognathic surgery.

## 1. Introduction

### 1.1. Orthognathic Surgery and Surgical Occlusion

Orthognathic surgery aims to correct skeletal abnormalities as well as to restore functional and aesthetic harmony to the maxillofacial region [1,2,3]. Aesthetic restrictions such as excessive gingival display, dental midline deviation or laterognathia can be treated with orthognathic surgery. Some functional aspects which involve dento-skeletal traits causing functional disturbance and/or occlusal trauma as well as severe obstructive sleep apnea can pose an indication for orthognathic surgery as well. These traits have been highlighted in the index of orthognathic functional treatment need (IOFTN) which helps to detect patients who need orthognathic surgery [4,5]. Once indication has been established, successful surgical outcomes rely on precise preoperative planning with dental occlusion serving as a critical determinant of postoperative stability and patient satisfaction [6,7,8]. Traditionally, final occlusion, also referred to as surgical occlusion, has been established through the use of dental stone casts and manual adjustments. This process relies on the haptic feedback of the occluding casts while the clinician inspects critical aspects such as dental midline, overbite, overjet, occlusal cant, presence of cross-bite, balanced occlusal contacts and overall stability of occlusion.

However, this process of establishing surgical occlusion has numerous challenges and disadvantages. Firstly, impression-taking can be uncomfortable in this group of patients who have orthodontic brackets on their teeth. Furthermore, the orthodontic brackets and wires introduce inaccuracies into the impression due to drags of the impression material. Secondly, the manufacture of dental stone casts from these impressions is time-consuming and prone to inaccuracies due to shrinkage of the impression material. Finally, the dental stone casts are brittle and poorly resistant to abrasion [9]. Thus, some occlusal details are lost during the manual setting of final occlusion.

### 1.2. Virtual Surgical Planning

Virtual surgical planning (VSP) has been well-established and popularized for orthognathic surgery due to its undeniable advantages over conventional planning by enabling three-dimensional (3D) visualization of the face, simulation of surgical movements and high accuracy of plan-to-patient transfer [10,11,12,13,14]. In VSP, the final occlusion has to be digitalized before any virtual surgery and simulation can be carried out. Many surgeons today still rely on the traditional method of manually setting the final occlusion using dental stone casts, which is then digitalized using surface scanning. This method is time-consuming, not cost-effective and inadvertently introduces errors as mentioned previously.

With the advent of intra-oral scanning and 3D printing, an intra-oral scan can now be used to replace traditional dental impressions while more resilient 3D-printed resin models replace traditional dental stone casts. One can then use these 3D-printed models to set the final occlusion before digitizing it using surface scanning or CBCT scanning. This method increases the accuracy of dental registration significantly [15,16]. However, utilizing 3D-printed resin models is much more expensive and still remains a time-consuming process. Moreover, in times of further globalization and digitalization fueled by the recent SARS-CoV-2 pandemic, the manual method of setting final occlusion using either traditional dental stone casts or 3D-printed resin models may pose some inconveniences as it requires a transfer of physical models between orthodontist and surgeon.

With the advent of digital technology, virtual occlusion has emerged as a promising alternative to conventional methods [17]. This approach involves creating 3D digital models of the dentition in stereolithography (STL) file format obtained by either surface scanning of traditional dental stone casts or intra-oral scanning. The latter is preferred due to higher accuracy and time-efficiency. The STL file is then transferred to a VSP software which allows one to virtually set the final occlusion. However, the tools used to set virtual occlusion rely heavily on visualization on a computer screen as haptic feedback cannot be obtained in this method. 

### 1.3. Mixed Reality

Mixed reality (MR) is an advanced technology that introduces virtual objects into the field of view of the user to create a seamless blend of the physical and digital worlds [18,19]. Unlike in virtual reality (VR), the real world is still visible in MR. Similarly unlike in augmented reality (AR), virtual objects are not simply overlaid onto the real environment, but rather integrated into the real environment. This is enabled by spatial computing and sensor technology, which allows the MR headsets to see and understand the real-world objects. Users can then interact with and manipulate the virtual objects in real time. Mixed reality blends the real and virtual worlds providing immersive experiences where digital information overlays the physical environment. In the field of medicine, MR holds immense promise as it can provide surgeons with real-time patient data and visual guidance and has found diverse applications [20,21,22,23]. As it is compatible with the data sets of virtual surgical planning, surgeons can already benefit from MR. During preoperative planning, MR allows the surgeons to visualize 3D models of a patient’s anatomy, aiding in surgical precision and reducing surgical risks. Among other application examples for maxillofacial surgery, this technology can help with surgical decision-making concerning the preferred mode of reconstruction in orbital trauma surgery and can serve as an immersive communication platform for interdisciplinary exchange during multidisciplinary tumor boards [24,25]. Surgical training can be greatly enhanced through realistic and interactive simulations and immersive learning experiences [26,27]. Moreover, MR can assist in medical education, enabling students to explore anatomical structures in a lifelike manner [28,29]. Additionally, MR can be utilized as a patient education tool to present medical information in a more accessible and engaging manner, facilitating better understanding and informed decision-making [30]. With its potential to revolutionize medical practices, mixed reality stands at the forefront of innovation, offering a transformative approach to enhance patient care, training and research.

This paper describes the first fully functional prototype tool to set final occlusion during computer-assisted orthognathic surgery planning utilizing mixed reality.

## 2. Methods/Results

### 2.1. Hardware: Magic Leap 1

**Figure 1:**
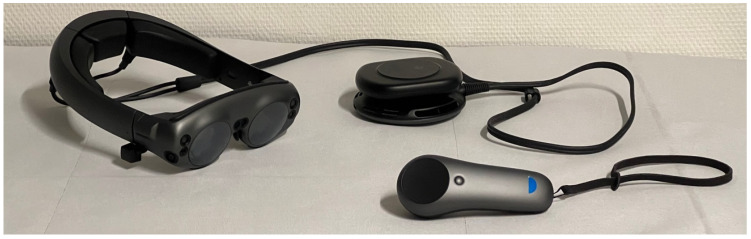
**Hardware components of Magic Leap 1.** The Magic Leap 1 mixed reality device consists of a headset connected to a light-pack and a separate controller. The semi-transparent glasses of the headset allow the user to see the real-world environment while virtual objects are projected into the field of view of the user. Thanks to the sensor technology of the headset and the spatial computing of the light-pack, virtual objects are integrated into the real-world environment respecting the user’s free locomotion in space. The user can interact with the virtual objects via the separate controller allowing three modes of input (movement of controller along six degrees of freedom; tapping/swiping on touchpad; three buttons to push) and vibration as haptic feedback.

The Magic Leap 1 contains a MR headset connected to a light-pack and a separate controller (see Figure 1). Through the headset, virtual dental models are projected into the real-world environment. The light-pack is a small computation unit, which is separated from the glasses to make the headset lighter. The controller enables the interaction between the user and the virtual model. It contains a trigger button, a bumper button, a menu button and a touchpad. The position and orientation of the controller is tracked by the headset. This allows the user to interact with the digital model utilizing all six degrees of freedom (DoF).

### 2.2. Software: Brainlab Elements Viewer

**Figure 2:**
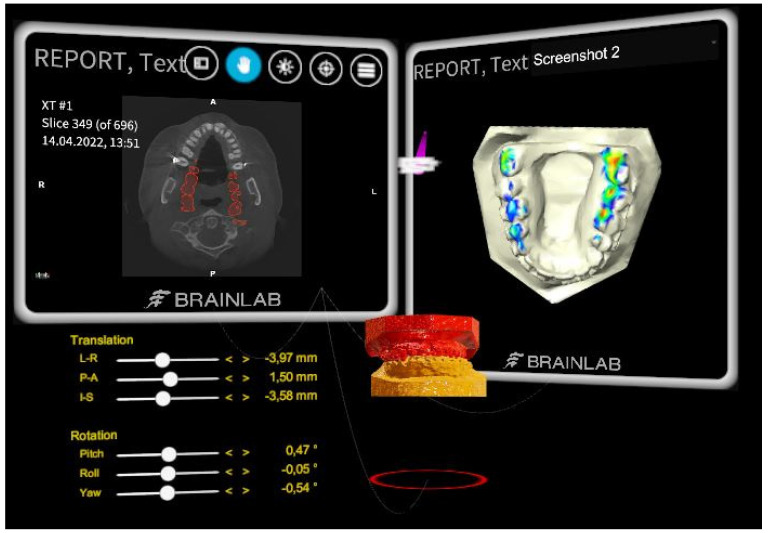
**Overview of the mixed reality working space.** Displayed are all virtual objects visible to the user against a black background which usually is occupied by the real-world environment during a planning session. The upper and lower virtual dental models (red and orange) are shown in the middle. On the lower left side, a movement table is displayed to quantify the spatial movement of the upper dental model in both translational and rotational aspects. It also allows small movement changes by ticking the arrowheads beside the desired axis. The red circle below the models indicates the presence of occlusal contact point(s) between the two models. This will turn green once their contact is relieved. In addition, a vibration in the controller is triggered when the occlusal contact is detected or relieved. An intersection map is displayed on the upper right side to illustrate the amount and intensity of occlusal contact point(s). In addition, other relevant information such as CT images can be displayed.

The orthognathic planning tool for virtual occlusion is built as a software prototype based on Brainlab’s Mixed Reality Viewer application (Brainlab Elements Viewer 5.3, Brainlab, Munich, Germany). STL files of the upper and lower dentition are imported into the desktop software and sent to the MR headsets. These STL files can be derived either (1) indirectly by (a) surface scanning dental stone models or 3D-printed resin models or (b) segmenting a CBCT/CT-scan of such dental models; or (2) directly by obtaining an intra-oral scan from the patient. For both procedures, the resulting STL files have to be digitally closed before importing them into the Brainlab software. This process can be described as digital “pedestaling” and involves obtaining watertight STL files with a manifold surface by using a 3D-modeling software, such as MeshMixer (Autodesk, San Francisco, CA, USA). The user then aligns the upper and lower dental arches manually via the controller input to achieve optimal occlusion. The real-time collision detection function helps notify the user when the virtual objects first collide (Figure 2).

### 2.3. User Interface and Interaction

**Figure 3:**
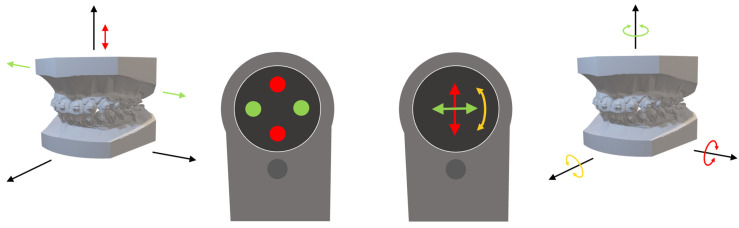
**Interaction commands.** The controller of the Magic Leap 1 allows three different modes of interaction: controller movement with six degrees of freedom (6DoF), tapping/swiping on the touchpad with the thumb and pushing of three buttons (trigger and bumper button on the front side and multi-use button on the top side beneath the touchpad). While the upper dental model can be grossly positioned using the trigger button with the index finger and moving it with free-hand movements in 6DoF, further fine tuning of movement can be achieved using the touchpad with the thumb. Tapping on the left/right axis (green circles) moves the model to the left/right or back/front depending on the user’s viewing angle. Tapping the up/down axis (red circles) will move the model up/down. Swiping across the touchpad as indicated (green, red and yellow arrows) will enable rotational movements which also are dependent on the user’s viewing angle.

The software prototype allows the user to freely move the upper dental model in 6 DoF via the MR controller, mimicking manual manipulation in real life. Additionally, fine adjustments can be made using the touchpad on the controller. Tapping the touchpad allows the user to translate the model by 0.1 mm selectively along any of the three axes (see Figure 3). For example, when standing in front of the model (coronal view), a tap on the left side of the touchpad moves the model to the left. The definition of the directions, however, is relative to the user, instead of to the model. Thus, when viewing the models from the side (sagittal view), the left–right command changes to front–back. This prevents unintended movements of the object and mimics real-life manipulation of the model. Swiping on the touchpad will rotate the model 0.1° around the yaw-, roll- and pitch-axis of the model.

**Figure 4:**
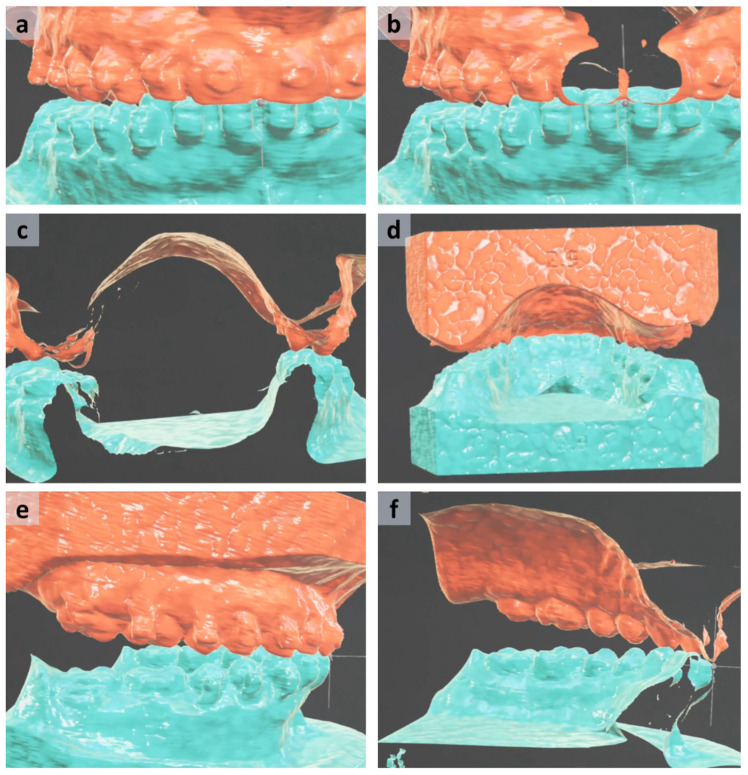
**Perspectives and cross-sectional views seen by the user of the Magic Leap 1 during the planning process of virtual occlusion setting.** By simple locomotion around and stepping through the virtual models, the user can easily change the perspective and create sectional views. The frontal view (**a**) and stepping through the models from the front (**b**) allow a precise assessment of the dental midline. Further locomotion from the front to the posterior regions of the dental arch enables inspection of the posterior tooth area on both sides for bilateral balanced occlusion contacts (**c**). Views from the rear (**d**) and from the sides (**e**) are easily achieved by approaching the models from the rear or from the sides including the sagittal sectional view (**f**) by stepping through the models from the side for evaluation of the correct overjet and overbite. See also Video S1 in Supplementary Material.

The user can walk around the models freely in order to inspect them from the rear, side or front. With the option of scaling the entire models, small details of the virtual objects can be inspected by the user which might be overlooked on a 2D display or in the physical world with normal-sized plaster models. Stepping through the models will automatically create a cut-through view with a distance of approximately 35 cm from the glasses. This allows a real-time section plane view of the models in sagittal, coronal or any desired orientation depending on the user’s position relative to the models (see Figure 4). By following the dental arch, the cross-sectional view results in an oblique sagittal/oblique coronal plane, allowing evaluation of occlusal contacts as well as overbite/overjet of every occluding dental units. This feature greatly enhances the inspection of the final occlusion which is impossible in the real-world environment.

### 2.4. Collision Detection

Collision detection is implemented to compensate for the absence of haptic feedback. It is performed every 30 ms, which can provide real-time information of interferences between the upper and lower dental models during the planning process. When a collision is detected, a visual indicator is provided in the mixed reality scene and a vibration in the controller is triggered.

Real-time collision detection of a complex model is a well-known problem due to the large number of vertices and faces. The brute force method would test every pair of faces in the model to determine the intersection between two models. This would result in the complexity of O(M*N), where M and N are the number faces of each model. Tree structure is often used to simplify the searching in space and improve the computation efficiency. Here an axis-aligned bounding-boxes (AABB) tree-based algorithm is implemented. This reduces the computation by excluding faces which are far away from each other from the actual collision detection process, which can be easily performed by comparing the bounding box of those faces without arithmetic calculation [31,32,33].

Therefore, the whole process of collision detection for the orthognathic planning tool contains two parts: (1) AABB tree build followed by (2) collision test with (a) broad and (b) narrow phases [31,32,33].

**Figure 5:**
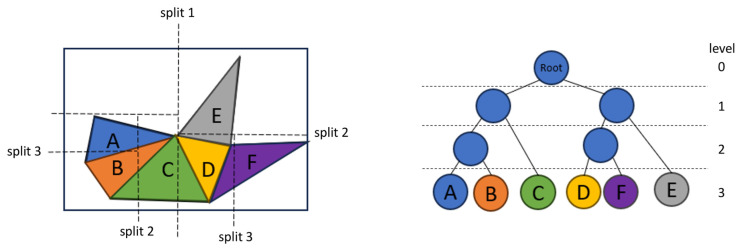
**AABB tree building process.** For explanation reasons, in this example 3D complexity has been simplified to a 2D object consisting of 6 faces A–F shown on the left side. The bounding box of the whole model (equal to‚ Root on level 0 in the AABB tree on the right side) is split along the dashed split line 1 which separates the two bounding boxes at level 1 consisting of the faces A–C and D–F, respectively. Further iterative splitting of the 2D space along the dashed split lines 2 and 3 leads to bounding boxes which consist of only one face each at level 3. This marks the end of the recursive AABB tree-building process resulting in the internal AABB tree data structure on the right side which serves for the collision test.

**Figure 6:**
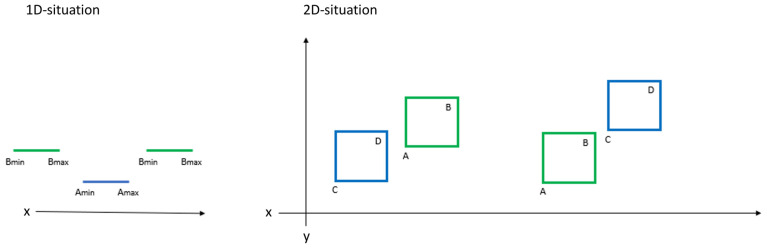
**Collision test in one dimension (1D) and two dimensions (2D).** To simplify the complexity of the 3D collision test, a 1D situation is displayed to explain the basic principle (see left side). In the 1D situation, the bounding boxes are degenerated into 1D intervals: [A_min, A_max] and [B_min, B_max]. To test if two intervals A and B are intersecting, A_min and B_max (or A_max and B_min) are compared. There is no intersection if B_max < A_min or B_min > A_max. In a 2D case (see right side), a bounding box can be characterized by the lower-left corner (with coordinates A_x, A_y) and top-right corner (with coordinates B_x, B_y). To test the intersection of two bounding boxes (A_B and C_D), the comparison can be performed on each axis. If A_x > D_x or A_y > D_y or B_x < C_x or B_y > C_y, then the two bounding boxes have no overlap. Similar tests can be extended to a 3D situation.

1. AABB tree build: In an AABB tree, bounding boxes aligned with axes are created to divide the spaces into blocks and each block contains one or more primitives (Figure 5). Two AABB trees are created, one separate tree for each of the dental models. The two AABB trees are created immediately after the data are loaded. A recursive way is taken to build the AABB trees, starting from the bounding box of the whole model (level 0). A split axis is selected and a split plan is calculated. Then the bounding box is split into two and all the primitives are partitioned into two groups according to the split plan (level 1). This process is recursive, building multiple levels of bounding boxes which contain fewer primitives as the degree of levels increases. The recursion stops when the bounding boxes of the last level contain only one primitive each, in this case one face.
2. Collision test: The collision test is performed every time the upper model has been moved. It compares the vertices of the upper and lower dental models for collision using the two AABB trees which have been built immediately after data-loading. It can be divided into (a) broad and (b) narrow phases while both follow the same basic principle (see Figure 6).
  - In the broad phase, bounding boxes of level 1 of the two AABB trees for each of the dental models are checked for intersection as explained above. All bounding boxes of level 1 (together with all the primitives they include) that are not intersecting are excluded from further collision testing. In the case of orthognathic planning, most vertices are excluded through this simple test and this greatly improves the efficiency of calculation.
  - When two bounding boxes are intersected, a narrow test is performed to determine the penetration depth of each vertex. The penetration depth is calculated as the signed point-to-plane distance between each vertex in object A and the plane defined by the triangle in object B to generate the intersection map of object A. If the vertex is inside object B, the penetration depth is positive and otherwise negative. The intersection map of object B is calculated vice versa.

### 2.5. Network Communication

The presented prototype is built on Brainlab Elements Viewer 5.3, ensuring a seamless integration into clinical workflows akin to the commercially available product. Patient data can be directly accessed from the Picture Archiving and Communication System (PACS) through the hospital’s internal network communication. This data can be viewed or processed using a desktop viewer. Incorporating the Mixed Reality Headset is facilitated by scanning a QR code, which is automatically recognized by the headsets. This QR code contains all necessary information to establish a wireless network connection between the headset and the server or workstation. The hardware accommodates conventional Wi-Fi connections. However, as part of the “5G.NRW–Giga for Health” research project, the implementation of certain Mixed Reality functionalities within a 5G/multi-access edge computing (MEC) infrastructure has been prototypically realized and tested. This was aimed at assessing the benefits of high bandwidth and low latency. Irrespective of the chosen network topology, multiple users can participate in a Mixed Reality session. This approach facilitates the amalgamation of expertise across various clinical disciplines. Users can either independently access the same data set or simultaneously engage in a multi-user session (e.g., orthodontist, maxillofacial surgeon, prosthodontist). This also enables technicians to prepare the data set, and physicians can approve the results even if they are not in the same space simultaneously.

### 2.6. Process of Setting Virtual Occlusion in Mixed Reality Environment

**Figure 7:**
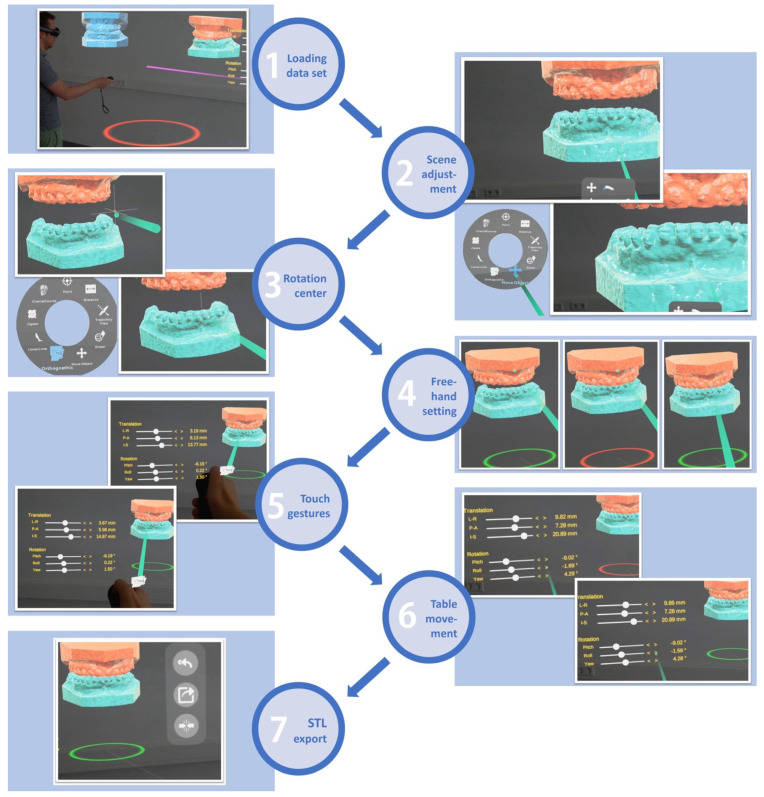
**Process of setting virtual occlusion in mixed reality environment.** Step 1: loading data set; Step 2: scene adjustment; Step 3: defining rotational center; Step 4: free-hand setting; Step 5: refinement using touch gestures; Step 6: fine-tuning using movement table; Step 7: STL export. Further details are explained in the text section process of setting virtual occlusion in mixed reality environment. See also Video S1 in Supplementary Material.

The process of setting a virtual occlusion for orthognathic surgery using mixed reality involves several crucial steps, each contributing to the precise planning and execution of the procedure. A detailed walkthrough is described below and depicted in Figure 7. The actual setting of the occlusion is adapted from the protocol of Ho et al. [34].

1. Loading Data Set:

The process begins with importing the patient’s data set, which includes the upper, lower dental models and the models in the starting occlusion. Other information such as the cone-beam computed tomography (CBCT) scans and skeletal models can be imported as necessary.

2. Scene Adjustment:

Subsequently, the virtual scene is adjusted to scale the models to an appropriate size and to position the occlusion plane of the lower jaw slightly beneath the viewing axis of the user. This position ensures that the occlusion adjustments are made in a realistic perspective, facilitating easy inspection and enabling free locomotion of the user around and through the virtual scene.

3. Tool Selection and Adjustment of rotational center:

The orthognathic tool is then chosen within the mixed reality environment. On each side, further interfaces occur to enhance information content:

On the right side, a movement table displaying the six degrees of freedom for both the translational and rotational movements is displayed. Values are set to zero defining the current position of the starting occlusion. The upper dental model can be moved using either hand gestures or tapping small arrow buttons for movements along the six axes in this movement table.

On the left side, an occlusal view onto the upper and lower dental arch is displayed visualizing occlusal relations via real-time heat map analysis.

Next, the rotational center needs to be defined to facilitate rotational movements. Typically, the lower dental midline is selected. In cases of missing lower incisor(s), another center can be chosen. This step fulfills two purposes: The marking of the lower midline facilitates the process of adjusting the corresponding upper midline by translational movements. Moreover, defining the rotational center at the dental midline enables rotational movements without changing the midline once it has been set by translational movements.

4. Initial Occlusion Setting by free hand movement:

The occlusion can first be set relating the upper and lower dental models grossly utilizing a free-hand approach, keeping in mind the raw midline, roll, yaw and pitch adjustments to achieve an approximate occlusion.

5. Touch Gestures for refinement:
  Finer adjustments of the occlusion can then be performed using touch gestures. This involves the following steps:
  - Correct the cant and midline of the upper model compared to the lower model in the frontal section view.
  - Set the overjet by viewing from the sagittal view. This step establishes the appropriate sagittal relationship between the upper and lower teeth.
  - Establish the overbite with translational-down movement while observing from the side with a highly pitched upper model to prevent collision in the molar region. This adjustment determines the vertical overlap between the upper and lower teeth in the front.
  - Balance the yaw rotation to establish a physiological intercuspation of the bilateral posterior dental arch preventing a cross-/scissors-bite relationship in the molar region.
  - Reduce the pitch while re-leveling the roll and yaw angles, viewing the models from the rear. This action fine-tunes the occlusion and ensures harmonious dental alignment leading to correct and maximum intercuspation.
6. Fine Tuning using Movement Table:

The above-mentioned movement table can be used for additional finer adjustment. In this phase, an asymptotic approach to a state of almost-collision is useful which means minimizing the distance between the models by 0.1°/0.1 mm steps before the first collision occurs. During this process, collision is intuitively indicated by haptic feedback through vibration of the hand controller and by color change of the ring beneath the virtual dental models. This last step maximizes intercuspation of the set occlusion, finalizing the process of setting virtual occlusion.

7. STL export

The occluding pair of dental models can now be exported as one STL file by the click of a button. The exported STL file can be implemented as the final occlusion in any commercially available orthognathic planning software without the need of further data post-processing.

By combining these seven steps, the process of defining a virtual occlusion using mixed reality for orthognathic surgery becomes comprehensive, convenient and precise. This approach ensures that the virtual occlusion closely mirrors the intended surgical outcome, contributing to successful surgical planning and execution.

The learning curves are steep, given the intuitive process facilitated by real-world integration via mixed reality. After five to ten cases (depending on prior experiences with mixed reality environments), case planning yields reproducible results within a reasonable timeframe. The overall time for this procedure takes less than 10 minutes. In cases with harmonious dental arches and good defined intercuspation, the process is more straightforward and takes an approximate duration of less than 5 minutes.

## 3. Discussion

**Figure 8:**
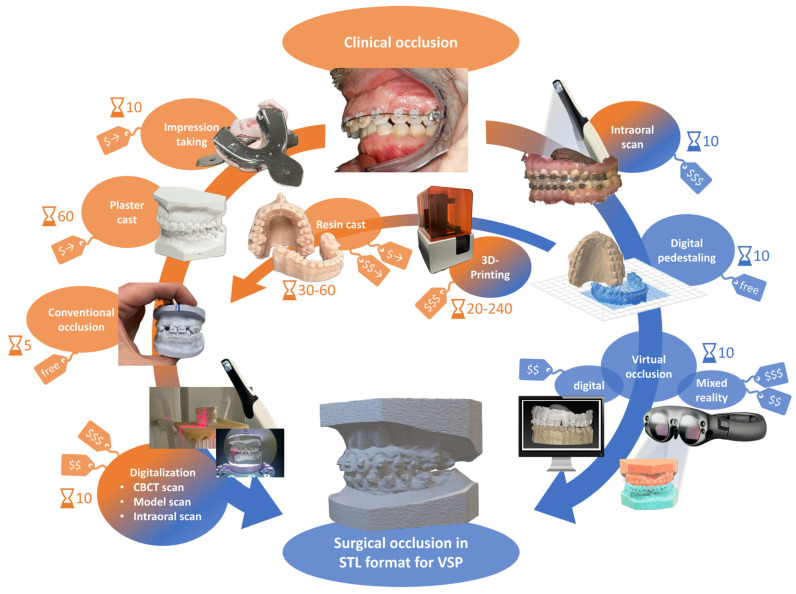
**Workflow paths from clinical starting occlusion to surgical occlusion.** The starting point is the real patient with his/her clinical occlusion after orthodontic treatment and before orthognathic surgery. The figure shows different pathways of setting the surgical occlusion and digitizing it to create a digital STL file format which is needed for utilization of virtual surgical planning (VSP). Depicted is the conventional method with dental stone models on the left side which mainly takes place in the physical world (orange). The virtual occlusion setting is depicted on the right side which enables a fully virtual workflow (blue) after intra-oral scanning using either a 2D computer screen or mixed reality device. A crosslink between the two paths via 3D printing still allows for the conventional method of occlusion setting while enabling the advantages of intra-oral scanning such as high accuracy. The average time needed for every single step is shown next to the hourglass symbol in minutes. Price tags indicate low ($), medium ($$) or high ($$$) costs as a one-time purchase while ongoing cost are indicated with →.

In the realm of orthodontics and orthognathic surgery in connection with the well-established virtual surgical planning, the advantages of virtual occlusion over the traditional cast-based approach are becoming increasingly evident, as illustrated in Table 1. An overview of the different workflows is depicted in Figure 8. The conventional workflow takes about 90 minutes from the clinical starting occlusion to the virtualized surgical occlusion. Compared to this, a full virtual workflow takes a third of the time with only 30 minutes. If a combination of workflows of the physical and virtual world is desired via 3D printing, time efforts are comparable to the plaster cast method or are increased depending on the material and devices used for 3D printing. Regarding costs, exact figures are challenging to determine as the current prototype is not commercially available. However, a crucial component is the software running the system, accompanying the hardware device. The full potential of the set-up is only unleashed when the system is used in conjunction with an intra-oral scanner, totaling the approximate costs in the middle four-digit range. Therefore, the conventional method appears more cost-effective as there is only one cost-intensive device needed for digitalization (CBCT, model scanner or intra-oral scanner). However, there are fewer ongoing costs in the virtual pathway, potentially leading to immortalization with a sufficient number of cases over time. Additionally, as mentioned earlier, time savings must be considered. Moreover, there may be future uses for a mixed reality device, enhancing its versatility as a tool for clinicians with a broader range of application possibilities and lowering the costs for purchase.

In addition to time and consumable savings, virtual occlusion facilitates telemedicine, enabling the seamless sharing of treatment plans among collaborating specialists [35]. These benefits extend to dental monitoring, enabling patients to utilize smartphones for home scanning of their current dentition. The scanned data can then be shared via cloud services, facilitating close follow-ups without the necessity for the patient’s physical presence within the practice. This introduces new dimensions of efficiency during extended treatment phases and even post-treatment follow-ups [36,37]. 

Nevertheless, it is crucial to recognize the limitations of virtual occlusion, especially the lack of haptic feedback present when manipulating the dental models manually. This tactile element appears to be vital in achieving a stable occlusion with the maximum amount of intercuspation [38]. Thus, in the transition to virtual occlusion, the challenge of replicating this sensory feedback must be navigated.

A fundamental aspect of orthodontics is the pursuit of a harmonious and functional occlusion characterized by factors such as midline alignment, correct incisal overjet and overbite, the relationship between the canine, Angle class I relationship and molar intercuspation with a maximum multi-point contact. These parameters serve as benchmarks for post-treatment assessment in the field of orthodontics and can be assessed using validated evaluation methods [39,40]. However, delving into the intricacies of orthognathic planning, a more complex reality is revealed because one has to accept that the ‘final’ occlusion for surgery planning will not be the final treatment occlusion due to the post-surgical orthodontic treatment phase. It is imperative to understand that achieving the ideal occlusion in orthognathic surgery is not always straightforward. Even with excellent pre-surgical orthodontic preparation, there regularly exists a bone-related malocclusion such as a transverse discrepancy of the upper and lower jaw bones. This intricacy results in the challenge of aligning two dental arches which often will not perfectly match in their current pre-surgical state, making it necessary to reconsider what constitutes the perfect or stable surgical occlusion. As such, a clinically acceptable occlusion for surgical planning has to be considered using other aspects of occlusion evaluation compared to final post-orthodontic states. While the desired occlusal stability for orthognathic surgery is mostly referred to as bilaterally supported occlusion with maximal intercuspation, a true definition of or guideline for mandatory occlusal features has not been established [41,42].

In practice, it is rare to achieve a perfect occlusion immediately after orthognathic surgery. In view of the emerging concept of the surgery-first approach, concepts of surgical occlusion are re-evaluated [43,44,45]. The surgery-first approach shifts a substantial portion of orthodontic treatment to the post-surgical phase, thereby enabling an earlier correction of facial deformity with enhanced patient satisfaction and shortening of the overall treatment time [46]. This strategy leverages the enhanced bone metabolism, particularly on the osteolytic/catabolic state following osteotomy, which contributes to faster tooth movement [47]. Occlusal splints secure the early vulnerable phase and accelerated post-surgical orthodontic treatment allows swift correction of the occlusion. Therefore, there is an apparently lower need for stability of surgical occlusion in surgery-first protocol—which also is referred to as ‘interim transit malocclusion’. However, multiple studies tried to investigate features of occlusal relations defining the boundary between indication and contraindication for surgery-first protocol [44,48]. Liao et al. defined guidelines for surgical occlusion set-up in the correction of skeletal class III deformity using the surgery-first approach [45]. Following these guidelines, the authors found that five to six pairs of occluding teeth would be enough for stable surgical occlusion set-up, in which an occlusal contact was defined as interocclusal distance being 0.5 mm or less. In most cases, a distribution of occlusal contacts across three out of three segments was achieved while even contacts in only the anterior segment were sufficient. Liao et al. emphasized that either a deep bite or a posterior open bite is advantageous, as it is easier to be closed orthodontically up to a discrepancy of <10 mm and it leaves space for further orthodontic adjustments with regard to posterior cross bite and anterior decompensation.

In light of these considerations, the pursuit of maximal intercuspation cannot always be the ultimate goal in orthognathic surgical planning, even in cases following the conventional three-stage procedure of the pre- and post-surgical orthodontic treatment phase. On the contrary, intentionally locking the posterior bite can frequently be necessary to address skeletal discrepancies and avoid an anterior open bite. Deliberate three-point contacts may also be desired to maintain facial height and subsequently stabilize it through orthodontic refinement. Even cases of protracted preoperative treatments can lead to the orthodontic desire for an ‘early’ surgery (sometimes driven by impatience and personal appointment requests from patients) that compromises in the stability of surgical occlusion to a degree comparable to the surgery-first concept. 

These individual approaches highlight that the goal of orthognathic surgical occlusion is more nuanced than simply maximizing intercuspation by haptically pressing two models on top of each other until there is no instability. This paradigm shift challenges the conventional belief that immediate post-surgical occlusion stability is paramount [49,50]. In addition, modern concepts of osteosynthesis techniques offer robust retention that reduces the risk of early relapse [51,52,53]. Therefore, the perceived advantages of haptic feedback for the conventional dental cast-based method must be reevaluated within this context. From the authors’ point of view, the relation at the inter-canine region seems to be more important as post-surgical orthodontic correction in this region is limited. Therefore, the requirements of virtual occlusion should be focused on dental midline, overjet and overbite as well as the inter-canine relationship in terms of roll and yaw movements with stable occlusion focused in this area.

Despite these complexities and lack of well-defined guidelines concerning the set-up of surgical occlusion, several concepts of virtual occlusion have been developed and applied in clinical settings to meet the treatment requirements [17]. These concepts predominantly involve four approaches:

1. Free translational and rotational movement along the six degrees of freedom until the desired set-up is achieved
2. Setting of strategic occlusal contacts by spring-like connections followed by an algorithm which leads to an automatic shortening of the distance between the defined occlusal pairs to a minimum while still respecting a non-collision of the models
3. Using haptic feedback devices to mimic the conventional sensory experience
4. Fully automated algorithms to calculate clinically desired final occlusion

The authors describing these different approaches concluded that virtual occlusion is effective and clinically applicable [34,54,55,56,57,58,59,60]. However, there are some disadvantages that have been described. The last mentioned method (4) may not respect the at times very individual process of determining a final occlusion for orthognathic surgery [56,60,61] As explained above, the third method (3) is limited, as the devices used allow haptic feedback of the collision for only one spatial coordinate, rather than for a full network of coordinates that would be required for two occluding meshes of dental models [57]. The second method (2) principally works fine in well-prepared cases [54] and has been implemented in commercially available orthognathic planning software [55]. However, due to difficulty in arranging certain springs hierarchically, frequent deviation of important determinants such as the dental midline can occur in more complex cases, which must be corrected by more laborious post-processing. Thus, learning curves might be flat and the ‘spring’ method is usually combined with the first described method (1) which corresponds to the principle of the mixed reality prototype presented here. However, the authors who first described the ‘spring’ approach rightfully concluded that during virtual planning the surgeon has to ‘see’ not ‘feel’ the occlusion [54]. Mixed reality technology stands out as a groundbreaking development in the field of superior visualization. It offers an unparalleled 3D experience with complete immersion, enabling seamless perspective changes and real-time section views (see Table 2). These capabilities are unattainable in a physical world environment. While other methods such as head-tracked stereo glasses with 3D-like monitor-views combining haptic feedback devices have made strides in simulations of fracture repositioning in trauma cases [62], mixed reality technology takes visualization to an entirely new level.

Considering the rapid advancements in mixed reality across various disciplines and the modern applications it offers, this emerging method is exceptionally promising [24,25,63,64,65]. However, before widespread adoption, rigorous testing is required to assess its accuracy and clinical application. The future holds the potential for expansion, including the incorporation of an occlusion map and spring-like features for increased automation as well as segmentation of the dental arch for more complex surgeries such as segmental osteotomies. Ultimately, the full integration of the entire orthognathic planning algorithm into a mixed reality environment would encompass 3D representations of the skull and jaw movements, photorealistic face scans and soft tissue simulations. Such developments are poised to elevate computer-assisted planning to an unprecedented level of visualization, precision and comprehensiveness. Deeper development of the basic haptic feedback feature implemented in the current prototype may even enhance the presented method overcoming the often-discussed drawback of virtual occlusion setting compared with the conventional method.

Furthermore, this technology has the potential to revolutionize teaching methods and patient education. The immersive experience offered by mixed reality introduces a new era in computer-assisted planning, breaking the boundaries of traditional approaches and paving the way for a dynamic, interactive and highly informative platform.

## 4. Conclusions

With this new prototype, the evolution of orthodontic and orthognathic surgical planning continues to advance, driven by innovations like virtual occlusion and mixed reality technology. These developments challenge traditional notions of occlusion and treatment strategies, opening new horizons for precision, efficiency and patient engagement. As we step into this new world of computer-assisted planning, we anticipate transformative changes that will shape the future of orthodontics and orthognathic surgery.

## Figures and Tables

**Table 1:** Advantages of virtual occlusion in general compared to the conventional method.

No need for physical models	Less material cost Less personal effort Less lead time for model preparation and digitization after occlusal setting No tear and wear
No need for physical fixation of final set occlusion	Fixation material occlusal or vestibular can block line of sight for surface scanning or prone to artefacts in CBCT scanning in stone models More than 1 person may be needed / higher personnel expense for stone models Danger of injury with hot wax during fixation of stone models More likely to accept suboptimal result due to higher effort of repositioning dental stone models
Additional information can be visualized	Intersection map and occlusion map Skull model: effect of occlusion on skeletal discrepancy / facial proportions DICOM data
Suitable for telemedicine due to full digital data (Digitizing conventional model makes this still possible for conventional method but far more laborious)	No need for transportation of physical models with danger of loss or damage Online consulting for inter-disciplinar shared decision making during Virtual surgical planning Easy continuous reevaluation during pre-surgical phase of orthodontic treatment and also for post-treatment long-term follow-up possible Compatible with dental monitoring by patients Decreasing physical patient presentation Prevention of unnecessary patient presentation if occlusion is not ready for surgery
Advantages of conventional over virtual occlusion	Haptic feedback of occlusal contacts / occlusal stability No intersection possible

**Table 2:** Comparing digital methods of setting virtual occlusion with mixed reality method.

	Digital Setting of Occlusion	Mixed Reality Environment
Mode of visualization	2D monitor	Immersive 3D scene with free-floating objects
Display of information	Scaling of objects and display of multiple informations is limited to size of monitor	Nearly unlimited scaling and display of additional information as to 360° field around user
Interaction with scene	Using PC mouse as 2D-pointer device	6 DoF controller with touchpad and haptic feedback
Changing of perspective	○ Using same input device as for interaction such as PC mouse ○ For advanced users keyboard shortcuts	○ Intuitive by free locomotion around 3D object ○ Moving of entire scene with hand controller possible
Multi-user compatibility	Display can be shared via conference calls with screen sharing options	True multi-user experience with free-to-all interaction

## Data Availability

Data are contained within the article and Supplementary Materials.

## Supplementary Materials

The Supplementary Video S1 is shared and available online at: https://zenodo.org/doi/10.5281/zenodo.10072931, Video S1: Working steps and features of setting virtual occlusion in mixed reality environment.
